# Annotations

**Published:** 1909-07-10

**Authors:** 


### ANNOTATIONS.

### Graduated Work for Consumptives.

To every medical man who takes an interest in the progress of practical therapeutics and watches the trend of modern research, the system of graduated exercises and work first introduced and extensively tested by Dr. Paterson, of the Frimley Sanatorium, has been known by repute for some time. To the public at large, excluding those patients who have actually experienced it and their relatives, it was probably quite unknown until the report appeared in the newspapers of Dr. Paterson's lecture on this subject at the Conference on Tuberculosis, held last month in the Whitechapel Art Gallery. Graduated work, apart altogether from its beneficent effect as a therapeutic measure —which may be due to the promotion of auto-inoculation, as Dr. Inman believes—has aspects of a less strictly technical and medical kind, and on those aspects the lecturer laid a particular stress, which was by no means greater than they deserved. He quoted very aptly the aphorism of Dr. Kingston Fowler, that "a fool never recovers from consumption"; an aphorism which, if not invariably true, contains a most important warning and lesson. For it is the active co-operation of the patient and his own intelligent comprehension of and assistance in the sanatorium regimen which is vital and essential to the success of that form of treatment of tuberculosis. Physical improvement has indeed a psychological effect, as Dr. Paterson has said, and it is hardly less true that psychological well-being influences the results of physical therapeutics. Above all, graduated work tends to prevent the lethargy and laziness which has proved the ruin of many labouring men restored to health by sanatorium treatment; and by labouring men we do not mean those only who labour with their hands. Another important point is that the work performed should be of some practical utility, and not a mere ploughing of the sands; and yet a further advantage, of both medical and sociological importance, is that the discharged patient is fit to resume his employment the moment he returns to his home, thus avoiding the risks of insufficient nutrition due to penury. As an example of a combination of sound therapeutics with practical economics, Dr. Paterson's system is hard to beat.

### Lord Strathcona and McGill University.

Dominion Day, as July 1 is known in Canada, has this year been celebrated by an act of munificence on the part of Canada's grand old man, Lord Strathcona, towards the McGill University at Montreal, and especially towards the medical school therein. The benefaction consists of a sum of £100,000, of which it is stipulated that nine-tenths is to be devoted to the completion of the medical faculty buildings now in course of reconstruction, while the remaining £10,000 is to go towards the fund for the augmentation of the salaries of professors. Lord Strathcona is Chancellor of McGill University, and hence may be supposed to have an especial affection for that foundation; but it must not be overlooked that this is only one of a long series of educational endowments in Canada, for, amongst other things, he founded, built, and endowed the Royal Victoria College for Women. His last princely donation is not his only one, even to the McGill Medical School, for he long ago founded the chairs of hygiene and pathology, and in 1907, after the engineering and medical schools had been destroyed by fire, he presented a site and £60,000 for the replacement of these schools. With this sum the authorities projected a building not merely worthy of Montreal and Canada as they stand to-day, but designed to maintain a prominent place in the future greatness of the Dominion. With a confidence which speaks volumes for Canadian ideals and standards, a building was started, the total cost of which was to be at least £150,000, and it is the residue of this sum which has now been provided by the generosity of the Chancellor. McGill University has already a most honourable history, especially as regards its medical faculty, and there can be little doubt that, thus supported and endowed, it will long retain its proud position of the premier university of Canada, and its successful rivalry with the sister corporations within the territory of the United States. Placed in the centre of the French-speaking population of Canada, it has yet attracted teachers and pupils, not only from its own province, and from the rest of the Dominion, but also from over the border, and even from over the Atlantic. The practical munificence of Lord Strathcona can only have the effect of widening the usefulness of his university, an object which commands the sympathy of the medical profession in Britain as much as in Canada itself.

### The Preservation of Bodies.

Dr. Collingridge, medical officer of health for the City of London, announces in his last monthly report that Dr. Reichter's apparatus for the preservation of corpses has now been installed, and is in working order at the City Mortuary in Golden Lane. The body of an unknown man, which had been found drowned, was brought to the mortuary on March 10, and after a post-mortem had been made, it was kept in the apparatus from March 16 until April 29, when it was removed to the mortuary. Up to the date of the report the body remained in perfect condition for identification. Having regard to the fact that a month is the limit of time for which it is necessary to preserve bodies for this purpose, this practical test shows that Dr. Reichter's apparatus is most satisfactory and fulfils all requirements. The temperature during the seven days' trial varied greatly, the highest recorded being 73deg. Fahrenheit during the last week in May. The apparatus consists essentially of an airtight case in which a saturated solution of formic aldehyde is allowed to evaporate, and the air meanwhile is kept in circulation by means of a small fan. In addition to the preservation of bodies for identification, the apparatus is well suited for the inspection of bodies by coroners' juries or by relatives. The cost of formic aldehyde amounts only to a few pence per day, and thus economy can be added to the merits of this valuable apparatus, which seems certain to meet a long-felt public want.

